# Advanced Research in Age-Related Macular Degeneration: Special Issue

**DOI:** 10.3390/biomedicines12020392

**Published:** 2024-02-08

**Authors:** Oyuna Kozhevnikova

**Affiliations:** Federal Research Center Institute of Cytology and Genetics SB RAS, Pr. Lavrentiev, 10, 630090 Novosibirsk, Russia; oidopova@bionet.nsc.ru

Age-related macular degeneration (AMD) is an eye disease that is the leading cause of irreversible vision loss in people over 55 years of age [1]. Due to the aging of the global population predicted in the coming decades, the number of patients with AMD is expected to increase significantly [2]. AMD is a disease with a complex nature, the development of which is controlled by many interacting genetic and environmental factors that determine the form, rate of disease progression, and response to therapy [3]. The pathogenesis of AMD is based on a decrease in metabolic and regenerative processes characteristic of aging, a violation of the microcirculation and structural organization of the retina, the prerequisites and mechanisms for the transition of which into a pathological process remain unclear [4]. Therefore, studies of the molecular mechanisms of AMD pathogenesis and new approaches to its early diagnosis, treatment, and prevention are urgently needed.

Late-stage AMD is distinguished by the neovascularization (wet type) or geographic atrophy (GA) of the retinal pigment epithelium (RPE) cell layer (dry type). There is growing genetic, experimental, and clinical evidence supporting an association between the pathophysiology of dry AMD and key proteins in the complement cascade. As a fundamental component of the innate immune system, the complement cascade protects the body from foreign pathogens and altered self-tissues. This highlights the crucial role that inflammation plays in the onset and progression of the disease. In a review, Patel et al. [5] provide an understanding of the role of the complement system in dry AMD and discuss emerging therapies in early-phase clinical trials. It is hoped that these drugs could potentially intervene early in the pathogenesis of dry AMD, halting the progression of the disease to a later stage. This pathway provides an abundant target for the synthesis of novel therapeutics. One of them—pegcetacoplan, additionally denoted as APL-2—is a PEGylated peptide inhibitor of C3 formulated by Apellis Pharmaceuticals that is administered intravitreally [6]. Moreover, on 17 February 2023, the Food and Drug Administration approved Syfovre^®^ (intravitreal pegcetacoplan, 15 mg, Apellis Pharmaceuticals, Waltham, MA, USA) based on a slower progression of atrophy in the DERBY and OAKS phase 3 randomized controlled clinical trials [7].

In a tutorial, Biarnés et al. [7] critically appraise the methodological aspects of phase 3 clinical trials in GA in relation to their design, analysis, and interpretation. The authors reviewed some of the key methodological attributes of phase 3 clinical trials in GA available in the main public registry of clinical trials as of 20 May 2023 (GATHER1, DERBY/OAKS, CHROMA/SPECTRI, SEATTLE, and GATE) to improve their interpretation. The topics discussed included types of endpoints, eligibility criteria, *p*-value and effect size, study power and sample size, the intention to treat principle, missing data, the consistency of results, efficacy–safety balance, and the application of results [7]. While these clinical studies share many outcomes and general eligibility requirements, there are several aspects that might affect outcomes which are discussed in this work.

The quantitative autofluorescence (qAF8) level is a presumed surrogate marker of lipofuscin content in the retina [8]. Chandra et al. [9] investigated changes in qAF8 levels in non-neovascular AMD in a prospective cohort study. The AMD categories were graded using both the Beckman classification and multimodal imaging, allowing for the deep phenotyping of AMD. A total of 353 eyes were analyzed, representing the largest study of qAF8 in AMD to date. The results showed that qAF8 is decreased during the early progression of AMD. qAF8 levels seem related more to the loss of function and integrity of RPE cells rather than being due to abnormalities in the visual cycle [9].

Zhdankina et al. [10] assessed the retinoprotective potential of the JNK inhibitor 11H-indeno[1,2-b]quinoxalin-11-one oxime sodium salt (IQ-1S), using senescence-accelerated OXYS rats as a model of AMD. Retinopathy in the OXYS rats reproduced the major signs of dry AMD: dystrophic alterations to the RPE, thinning of the neuroretina, and impaired choroidal microcirculation [11]. Treatment with IQ-1S significantly improved the structural and functional parameters of RPE. This suggests that an increase in the JNK-signaling pathway may play a role in the treatment of dry AMD.

AMD shares some risk factors with diabetes mellitus, but little is known about the risk of DM in individuals with AMD. Jung et al. [12] investigated the association between AMD and the risk of diabetes mellitus using the Korean Nationwide Health Insurance Database. A Cox hazard regression model was used to examine the hazard ratios of diabetes mellitus with adjustments for potential confounders. The study did not find an increased risk of diabetes mellitus in individuals with AMD.

With the availability of high-resolution retinal imaging, the classification of macular neovascularization (MNV) and drusen has evolved. Optical coherence tomography (OCT) has improved our understanding of MNV types and has expanded and refined distinctions between drusen types. Subretinal drusenoid deposits (SDDs) have a high prevalence in AMD which was underestimated prior to OCT [13]. The purpose of the study by Muth et al. [14] was to assess the predictive odds ratios between drusen types and MNV lesion variants in patients with treatment-naïve neovascular AMD. Multimodal imaging was retrospectively reviewed for drusen type (soft drusen, SDD, or mixed) and MNV type according to CONAN criteria [15]. The authors concluded that SDDs represent a common phenotypic characteristic in AMD eyes with treatment-naïve MNV. The adjusted odds ratio for eyes with predominant SDDs to develop MNV 2 and MNV 3 was much higher, possibly due to their location in the subretinal space.

One study [16] aimed to report the clinical and multimodal imaging findings of patients with neovascular AMD who developed bacillary layer detachment (BALAD). This finding is not commonly described in the literature. BALAD is a definition introduced in 2018 to describe an OCT finding in a patient with Toxoplasmosis chorioretinitis and pachychoroid disease [17]. It is characterized by a splitting of the photoreceptor layer at the myoid level, resulting in a space posterior to the external limiting membrane.

Acquired vitelliform lesions (AVLs) are associated with a large spectrum of retinal diseases, including AMD [18]. The purpose of the study by Damian et al. [19] was to characterize AVLs in AMD patients using OCT and ImageJ software (version 1.53T). A vitelliform lesion represents an accumulation of lipofuscin, melanosomes, melanolipofuscin, and outer segment debris in the subretinal space [20]. The accumulation of subretinal fluid associated with vitelliform lesions was explained by a mechanical displacement between the RPE and outer retinal layers, which consequently inhibits the RPE from pumping out liquified lipofuscin debris [18,19]. The authors found increased RPE thickness as a sign of hyperplasia, contrary to the ONL layer, which was decreased, mirroring the impact of the vitelliform lesion over the photoreceptors. Most of the analyzed eyes presented a discontinuous ellipsoid zone. During follow-up, some of the AVLs had increased while others had regressed, which was demonstrated in divergent results between eyes regarding height, width, area, perimeter, or volume [19].

Neovascular AMD patients with alternative alleles of the *STAT4* genes rs10181656 and rs10168266 exhibited significantly lower serum STAT4 levels than the control group, suggesting a link between specific *STAT4* genotypes and serum STAT4 levels in AMD patients [21].

The first-line drugs for neovascular AMD are inhibitors of VEGF, with up to 30% of patients having an incomplete response to treatment [22]. Genetic factors may influence the response to anti-VEGF therapy and explain treatment outcome variability. Recently, autophagy was implicated in causing tumor resistance to antiangiogenic therapy [23], which suggests an analogous connection between autophagy and anti-VEGF intravitreal injections during AMD treatment. In this direction, a study was conducted assessing the role of polymorphic markers of autophagy genes in the risk of AMD and the anti-VEGF response [24]. It was shown that *MTOR* gene polymorphisms were moderately associated with neovascular AMD. The variants *SQSTM1*-rs10277 and *ULK1*-rs3088051 may influence the short-term response to anti-VEGF treatment. The results suggest that autophagy could be a target for future drugs to overcome resistance to anti-VEGF therapy [24].

Through published articles, this Special Issue provides new insight into the diagnosis and treatment of AMD and has the potential to impact current and future knowledge of the pathogenesis and treatment of patients with AMD. New diagnostic approaches and treatment regimens, the development of multimodal visualization methods and potential genetic and OCT markers of treatment response may help guide the future management of this disease.

## References

[1] Guymer R.H., Campbell T.G. (2023). Age-related macular degeneration. Lancet.

[2] Mitchell P., Liew G., Gopinath B., Wong T.Y. (2018). Age-related macular degeneration. Lancet.

[3] Fursova A.Z., Derbeneva A.S., Vasilyeva M.A., Nikulich I.F., Tarasov M.S., Gamza Y.A., Chubar N.V., Gusarevitch O.G., Dmitrieva E.I., Kozhevnikova O.S. (2022). New findings on pathogenetic mechanisms in the development of age-related macular degeneration. Vestn. Oftalmol..

[4] Telegina D.V., Kozhevnikova O.S., Kolosova N.G. (2017). Molecular mechanisms of cell death in retina during development of age-related macular degeneration. Adv. Gerontol..

[5] Patel P.N., Patel P.A., Land M.R., Bakerkhatib-Taha I., Ahmed H., Sheth V. (2022). Targeting the Complement Cascade for Treatment of Dry Age-Related Macular Degeneration. Biomedicines.

[6] Liao D.S., Grossi F.V., El Mehdi D., Gerber M.R., Brown D.M., Heier J.S., Wykoff C.C., Singerman L.J., Abraham P., Grassmann F. (2020). Complement C3 Inhibitor Pegcetacoplan for Geographic Atrophy Secondary to Age-Related Macular Degeneration: A Randomized Phase 2 Trial. Ophthalmology.

[7] Biarnés M., Garrell-Salat X., Gómez-Benlloch A., Guarro M., Londoño G., López E., Ruiz S., Vázquez M., Sararols L. (2023). Methodological Appraisal of Phase 3 Clinical Trials in Geographic Atrophy. Biomedicines.

[8] Sparrow J.R., Duncker T., Schuerch K., Paavo M., de Carvalho J.R.L. (2020). Lessons learned from quantitative fundus autofluorescence. Prog. Retin. Eye Res..

[9] Chandra S., Grewal M.K., Gurudas S., Sondh R., Bird A., Jeffery G., Chong V., Sivaprasad S. (2023). Quantitative Autofluorescence in Non-Neovascular Age Related Macular Degeneration. Biomedicines.

[10] Zhdankina A.A., Tikhonov D.I., Logvinov S.V., Plotnikov M.B., Khlebnikov A.I., Kolosova N.G. (2023). Suppression of Age-Related Macular Degeneration-like Pathology by c-Jun N-Terminal Kinase Inhibitor IQ-1S. Biomedicines.

[11] Kozhevnikova O.S., Telegina D.V., Tyumentsev M.A., Kolosova N.G. (2019). Disruptions of Autophagy in the Rat Retina with Age During the Development of Age-Related-Macular-Degeneration-like Retinopathy. Int. J. Mol. Sci..

[12] Jung W., Yoon J.M., Han K., Kim B., Hwang S., Lim D.H., Shin D.W. (2022). Association between Age-Related Macular Degeneration and the Risk of Diabetes Mellitus: A Nationwide Cohort Study. Biomedicines.

[13] Zweifel S.A., Spaide R.F., Curcio C.A., Malek G., Imamura Y. (2010). Reticular pseudodrusen are subretinal drusenoid deposits. Ophthalmology.

[14] Muth D.R., Toro M.D., Bajka A., Jonak K., Rieder R., Kohler M.M., Gunzinger J.M., Souied E.H., Engelbert M., Freund K.B. (2022). Correlation between Macular Neovascularization (MNV) Type and Druse Type in Neovascular Age-Related Macular Degeneration (AMD) Based on the CONAN Classification. Biomedicines.

[15] Spaide R.F., Jaffe G.J., Sarraf D., Freund K.B., Sadda S.R., Staurenghi G., Waheed N.K., Chakravarthy U., Rosenfeld P.J., Holz F.G. (2020). Consensus Nomenclature for Reporting Neovascular Age-Related Macular Degeneration Data: Consensus on Neovascular Age-Related Macular Degeneration Nomenclature Study Group. Ophthalmology.

[16] Palmieri F., Younis S., Raslan W., Fabozzi L. (2023). Bacillary Layer Detachment in Neovascular Age-Related Macular Degeneration: Case Series. Biomedicines.

[17] Mehta N., Chong J., Tsui E., Duncan J.L., Curcio C.A., Freund K.B., Modi Y. (2021). Presumed foveal bacillary layer detachment in a patient with toxoplasmosis chorioretinitis and pachychoroid disease. Retin. Cases Brief Rep..

[18] Juliano J., Patel S., Ameri H. (2021). Acquired Vitelliform Macular Degeneration: Characteristics and Challenges of Managing Subretinal Fluid. J. Ophthalmic Vis. Res..

[19] Damian I., Muntean G.-A., Galea-Holhoș L.-B., Nicoară S.-D. (2023). Advanced ImageJ Analysis in Degenerative Acquired Vitelliform Lesions Using Techniques Based on Optical Coherence Tomography. Biomedicines.

[20] Balaratnasingam C., Hoang Q.V., Inoue M., Curcio C.A., Dolz-Marco R., Yannuzzi N.A., Dhrami-Gavazi E., Yannuzzi L.A., Freund K.B. (2016). Clinical Characteristics, Choroidal Neovascularization, and Predictors of Visual Outcomes in Acquired Vitelliform Lesions. Am. J. Ophthalmol..

[21] Blekeris T., Gedvilaite G., Kaikaryte K., Kriauciuniene L., Zaliuniene D., Liutkevciene R. (2024). Association of STAT4 Gene Polymorphisms (rs10181656, rs7574865, rs7601754, rs10168266) and Serum STAT4 Levels in Age-Related Macular Degeneration. Biomedicines.

[22] Yang S., Zhao J., Sun X. (2016). Resistance to anti-VEGF therapy in neovascular age-related macular degeneration: A comprehensive review. Drug Des. Dev. Ther..

[23] Chandra A., Rick J., Yagnik G., Aghi M.K. (2020). Autophagy as a mechanism for anti-angiogenic therapy resistance. Semin. Cancer Biol..

[24] Kozhevnikova O.S., Fursova A.Z., Derbeneva A.S., Nikulich I.F., Devyatkin V.A., Kolosova N.G. (2023). Pharmacogenetic Association between Allelic Variants of the Autophagy-Related Genes and Anti-Vascular Endothelial Growth Factor Treatment Response in Neovascular Age-Related Macular Degeneration. Biomedicines.

